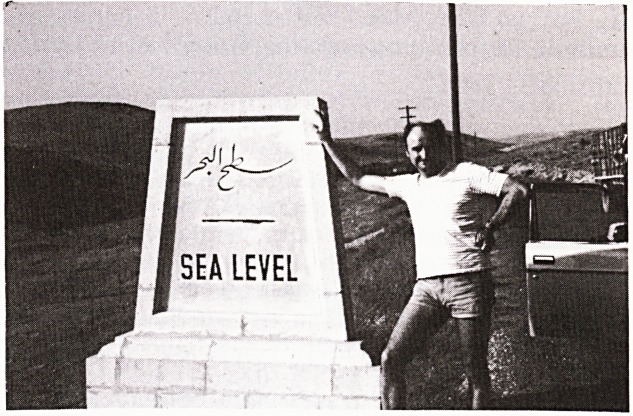# From Our Foreign Correspondent

**Published:** 1986-02

**Authors:** 


### From Our Foreign Correspondent

**Ammonites revisited**

Correspondents should clearly correspond. Regular and courteous are my postcards from the Editor inviting a contribution, but patchy (alas) in time and content has been the response. On this occasion he knew I had been to Jordan as an examiner. Craftily, to stimulate and pin me down, he sent a copy of his own first publication entitled 'On Transjordan – and surgery there'. This article appeared in the 1945 volume of the University of Leeds Medical Magazine. It is a charming account of 18 months spent as Surgeon to the Italian Hospital in Amman, the biblical city of the Ammonites and afterwards the Roman city of Philadelphia.

Much has changed of course from those halcyon days of the British mandate, though I dare say camel's urine may still be used to lacquer the hair and avert alopecia. Emir Abdullah is dead, assassinated in the Al Aksa mosque at Jerusalem in 1948. His grandson, who survived that attempt and others since, has proved to be a tenacious and energetic monarch. The mosque is now a part of Israel. Transjordan lost the 'trans' when it gained the West Bank from former parts of Palestine. Once more confined to the further shores of the river, Jordanians live more in hope than expectation of regaining their lost territory. The Allenby Bridge – faint echoes of the Raj – has become a difficult frontier for anyone to cross, especially Palestinians; yet cross it they must if they wish to see relatives and friends who have stayed behind.

I had visited Amman once before on the way home from another tour of examining in Iraq. On that occasion it had been pleasant to exchange the dusty plains of Mesopotamia for the hills and relative greenery of Northern Jordan. Michael Wilson obviously felt the same in coming up from Sarafand in Palestine (that takes some finding on the map). And what of the Italian Hospital? I am sorry to say I do not know, but I expect it is still standing. I did notice a small Italian Hospital in Karak. Here the Crusaders built a castle deep in the hills of Moab and overlooking the Dead Sea. It was defended successfully against Saladin in 1183 but subsequently fell to his brother. Is it true that Saladin could cut a cushion with his (curved) sword, while Coeur de Lion (what a disastrous king) could cut an iron bar with his broadsword? It seems that Saladin was much the better general.

Nearby to Karak is the great chasm of Wadi Mujab. Further North lies Madaba, where there is a wonderful 6th Century mosaic on the floor of the local Catholic Church. It is a map of the World – that is the Eastern Mediterranean – with the Holy City of Jerusalem at the centre. A large fish is depicted swimming in the Jordan against the stream to escape the hostile waters of the Dead Sea (24 per cent saline). Actually the Jordan and the Rio Grande are the world's two most disappointing rivers, closely followed by the Avon at low tide.

'I looked over Jordan and what did I see?
A band of angels comin' after me'.

What I saw were greenhouses and tanks, for the Jordan valley is at once the most fertile part of the country and a sensitive military area. When you descend from Amman to the Dead Sea (or like the Good Samaritan from Jerusalem to Jericho) you pass the sea level marker, a truncated obelisk, and keep on dropping for another 400 metres. Notices in Arabic and English discourage photography, but I had an inane wish to be photographed leaning against the marker and my Jordanian hosts obliged. Just around the corner we were stopped at an army checkpoint and the camera was promptly confiscated. Voluble arguments ensued and to my surprise the camera was handed back with much handshaking. While I watched on the fringe and awaited the outcome I thought of Hilaire Belloc's Cautionary Tales and composed the following:

'Oh, my friends, be warned by me,
When visiting the Mortal Sea.
If you snap the obelisk
You'll put your camera at risk'.

The photograph came out quite well.

I took part in two consecutive examinations, visiting the Jordan University Hospital and the older Al-Bashier Hospital in Amman. Students are taught and examined in English but think and write notes in Arabic. I thought they contended very well with a foreign examiner and they gently educated him in the mysteries of hydatid and other indigenous diseases. I found lunch in the hospital canteen something of an ordeal but happily Ramadan arrived to resolve the problem. On the first evening of Ramadan I was driven some 70 miles from Amman, past Mafraq where Glubb Pasha built a camp for the Arab Legion, past hundreds of lorries trundling North to Syria and Iraq, past a sensational sunset (signal for breakfast) to a concrete house, four square in the desert, home of the father of a doctor working in Bristol. Four of us sat down to dinner of mansaf, two whole lambs cooked with rice. The room was simply furnished with cushions round the walls and a video camera; during dessert we could watch ourselves tucking into the lamb.

Examinations over, I was taken to Petra and Aqaba. From Aqaba you can see four countries: Jordan, Israel, Egypt and Saudi Arabia. The Saudis actually gave Jordan several kilometres of coast to improve their harbour facilities (nice coast it is too with a coral reef just offshore). The only precedent I could recall for such generosity was our ceding Monmouthshire to the Welsh.

On the way South we followed the line of the Hejaz Railway, built by the Turks in 1908 to carry pilgrims to Medina. Nobody who has seen the film of Lawrence of Arabia could forget the torrid time he gave the Turkish armoured trains. 'The Turks then nearly cut us off as we looted the train', he wrote, 'and I lost some baggage and nearly myself. My loot is a superfine red Baluch prayer-rug. I hope this sounds the fun it is'. Lawrence and his Arab irregulars captured Aqaba in July 1917. The film was shot in nearby Wadi Rum, and Omar Sharif's double now runs the Oriental Gift Shop in downtown Aqaba. 'I come from Bristol', I said, 'like Peter O'Toole' and bathed in vicarious glory as I bought my postcards.

Robin Williamson

## Figures and Tables

**Figure f1:**